# Supplementary material for: Impact of Physico-Chemical Properties of Cellulose Nanocrystal/Silver Nanoparticle Hybrid Suspensions on Their Biocidal and Toxicological Effects
Source: Nanomaterials (Basel). 2021 Jul 20;11(7):1862. doi: 10.3390/nano11071862 (PMC8308223; doi:10.3390/nano11071862)
Supplement: Supplementary file 1 [file nanomaterials-11-01862-s001.zip › nanomaterials-1282676-supplementary.pdf]

Supplementary Materials

Impact of Physico-Chemical Properties of Cellulose Nanocrystal/Silver Nanoparticle Hybrid Suspensions on Their Biocidal and Toxicological Effects

Dafne Musino [1], Julie Devcic [2], Cécile Lelong [2], Sylvie Luche [2], Camille Rivard [3,4], Bastien Dalzon [2], Gautier Landrot [3], Thierry Rabilloud [2,*], and Isabelle Capron [1,*]

[1] INRAE, Institut national de recherche pour l'agriculture, l'alimentation et l'environnement, BIA, Biopolymères Interactions et Assemblages, 44316 Nantes, France; dafne.musino@inrae.fr
[2] Laboratoire de Chimie et Biologie des Métaux, University Grenoble Alpes, CNRS, CEA, IRIG, CBM, UMR5249, 38000 Grenoble, France; devcicjulie@gmail.com (J.D.); cecile.lelong@univ-grenoble-alpes.fr (C.L.); sylvie.luche@cea.fr (S.L.); bastien.dalzon@cea.fr (B.D.)
[3] SOLEIL Synchrotron, L'Orme des Merisiers, Gif-sur-Yvette, 91192 Saint-Aubin, France; Camille.Rivard@Synchrotron-Soleil.Fr (C.R.); gautier.landrot@synchrotron-soleil.fr (G.L.)
[4] INRAE, Institut national de recherche pour l'agriculture, l'alimentation et l'environnement, BIA, TRANSFORM, 44316 Nantes, France
* Correspondence: thierry.rabilloud@cnrs.fr;(T.R.); isabelle.capron@inrae.fr(I.C.)

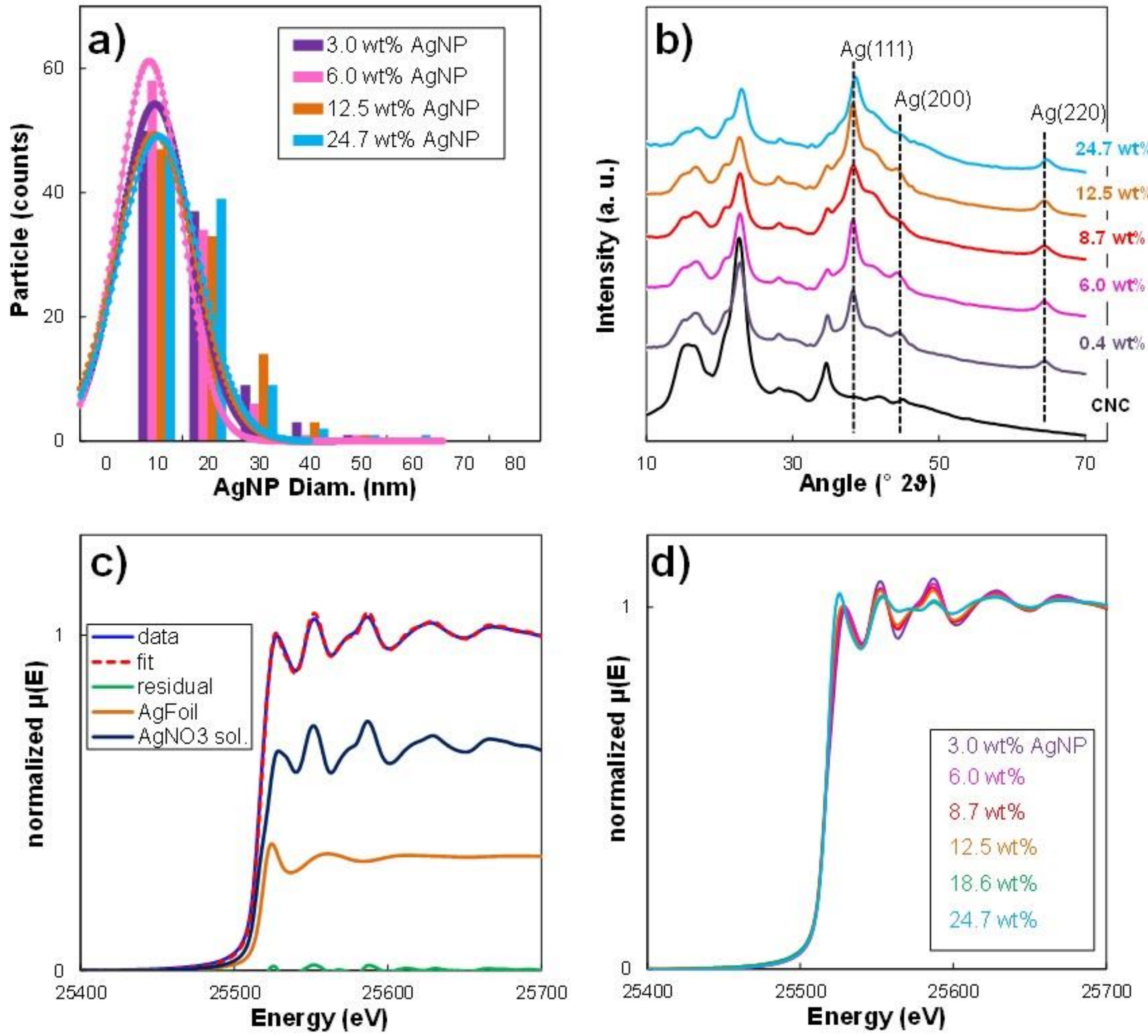

Figure S1. (a) Size distribution histograms of AgNPs in CNC/AgNP hybrids at various AgNP content (b) XRD diffractograms. (c) Example of a XANES spectrum and its corresponding linear combination fit (LCF) using Agfoil and $AgNO_3$ aqueous solution as components; (d) XANES data of CNC/AgNP hybrids at various AgNP content.

Table S1. R-factor and Chi-square values for linear combination fitting procedure applied to the XANES region of CNC/AgNP hybrid suspensions at different AgNP contents.

AgNP content (wt%)	R-factor	Chi-square	Ag_0 (%)[1]
**0.4**	-	-	-
**1.6**	-	-	-
**3.0**	0.0007987	0.02097	91 ± 9
**6.0**	0.001196	0.02775	75 ± 8
**8.7**	0.0007017	0.01885	65 ± 7
**12.5**	0.0003801	0.01034	57 ± 6
**18.6**	0.0003421	0.00949	32 ± 3
**24.7**	0.0002743	0.0074	34 ± 3

[1]the standard error is established as 10% of the measured value.

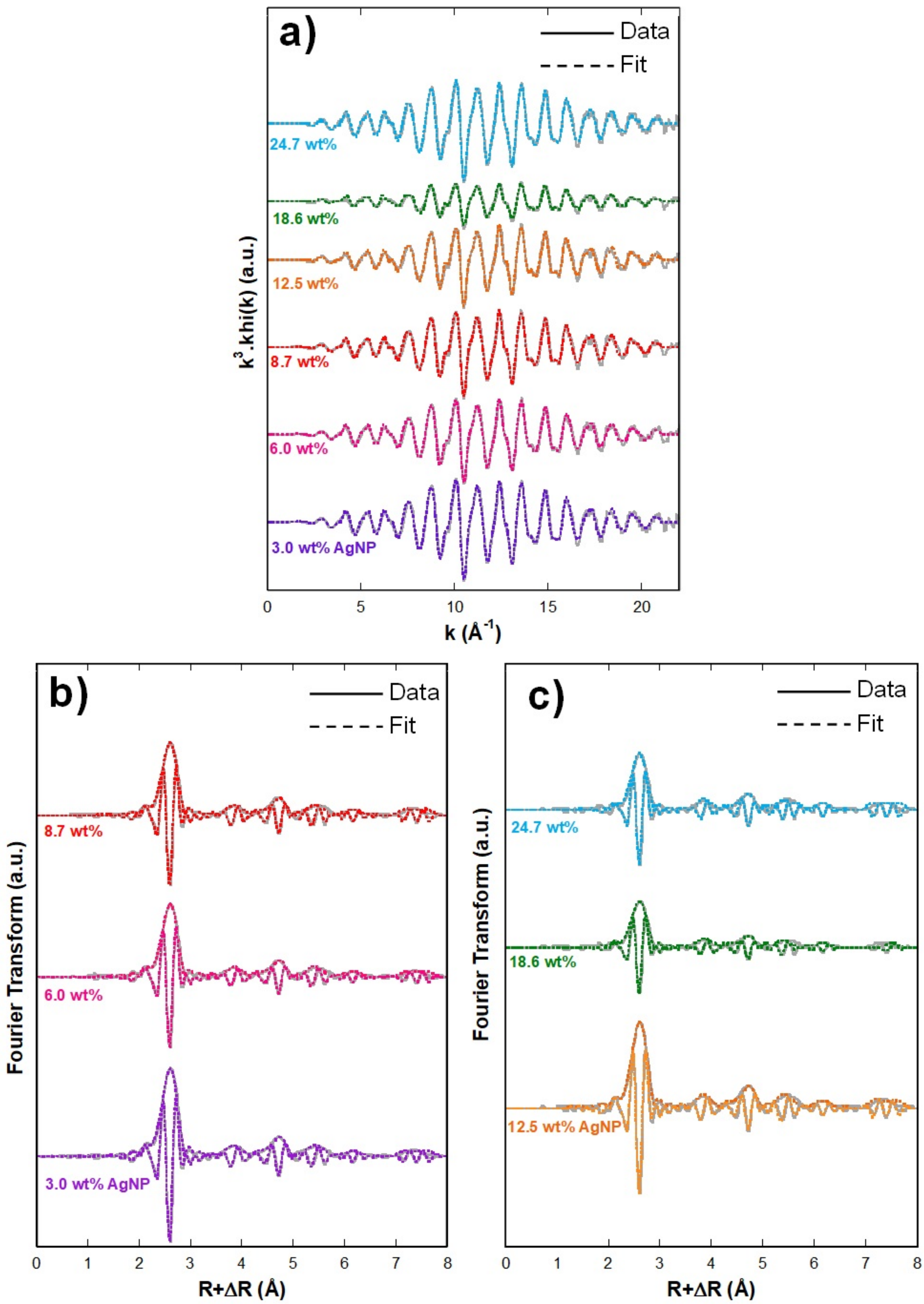

Figure S2. (a) EXAFS spectra Fourier transform (solid gray lines) and fit (dotted lines); (b) and (c) magnitude and imaginary part (solid gray lines) and fit (dotted lines) of the Fourier transform of hybrids at various AgNP contents.

Table S2. EXAFS fit results for CNC/AgNP hybrid suspensions with increasing AgNP content.

	Degeneracy of the paths						Debye-Waller factor σ^2						Variation in interatomic distance ΔR (Å)						Interatomic distance R (Å)					
	3.0 wt% AgNP	6.0 wt%	8.7 wt%	12.5 wt%	18.6 wt%	24.7 wt%	3.0 wt% AgNP	6.0 wt%	8.7 wt%	12.5 wt%	18.6 wt%	24.7 wt%	3.0 wt% AgNP	6.0 wt%	8.7 wt%	12.5 wt%	18.6 wt%	24.7 wt%	3.0 wt% AgNP	6.0 wt%	8.7 wt%	12.5 wt%	18.6 wt%	24.7 wt%
Ag1 ss	9.6±0.4	7.9±0.4	7.5±0.4	6.7±0.4	3.8±0.3	4.3±0.4	0.0038	0.0038	0.0036	0.0034	0.0036	0.0034	-0.031	-0.031	-0.030	-0.029	-0.030	-0.030	2.875	2.875	2.875	2.876	2.875	2.876
Ag2 ss	2.9±1.2	2.1±1.0	2.2±1.0	1.9±1.0	0.9±0.7	1.1±0.8	0.0041	0.0037	0.0035	0.0031	0.0028	0.0026	-0.037	-0.036	-0.037	-0.037	-0.033	-0.034	4.072	4.073	4.072	4.072	4.076	4.075
Ag1 Ag1 at	48*	48*	48*	48*	48*	48*	0.0057	0.0056	0.0054	0.0051	0.0054	0.0051	-0.046	-0.046	-0.045	-0.044	-0.046	-0.045	4.312	4.312	4.313	4.315	4.313	4.313
Ag3 ss	25.0±3.3	22.6±3.2	22.9±3.4	22.2±3.4	17.2±3.0	18.5±3.3	0.0066	0.0067	0.0066	0.0065	0.0073	0.0066	-0.035	-0.036	-0.036	-0.035	-0.036	-0.036	4.997	4.996	4.996	4.998	4.996	4.996
Ag1 Ag3 ot	96*	96*	96*	96*	96*	96*	0.0052	0.0052	0.0051	0.0050	0.0055	0.0050	-0.024	-0.024	-0.024	-0.023	-0.024	-0.024	5.398	5.397	5.398	5.398	5.397	5.398
Ag4 ss	4.6±3.7	5.6±3.5	5.6±3.9	6.3±4.0	15.8±2.2	18.7±2.2	0.0051	0.0053	0.0052	0.0053	0.0056	0.0046	-0.020	-0.014	-0.014	-0.013	0.031	0.045	5.791	5.797	5.797	5.798	5.842	5.856
Ag1 Ag4 fs	24*	24*	24*	24*	24*	24*	0.0089	0.0091	0.0088	0.0087	0.0093	0.0080	-0.050	-0.045	-0.044	-0.042	0.001	0.015	5.761	5.766	5.767	5.769	5.812	5.826
Ag1 Ag1 fta	12*	12*	12*	12*	12*	12*	0.0151	0.0150	0.0143	0.0137	0.0145	0.0135	-0.061	-0.061	-0.061	-0.058	-0.061	-0.060	5.750	5.750	5.750	5.753	5.750	5.751
Ag1 Ag4 Ag1 dfs	12*	12*	12*	12*	12*	12*	0.0089	0.0091	0.0088	0.0087	0.0093	0.0080	-0.050	-0.045	-0.044	-0.042	0.001	0.015	5.761	5.766	5.767	5.769	5.812	5.826
Ag5 ss	3.2±2.6	3.7±2.9	2.7±2.2	3.3±2.7	1.8±2.1	2.8± 2.6	0.0022	0.0027	0.0015	0.0020	0.0020	0.0021	-0.056	-0.060	-0.058	-0.057	-0.056	-0.059	6.441	6.437	6.439	6.439	6.441	6.438
Ag7 ss	36.9±8.7	44.6±14.2	35.2±8.1	33.9±7.2	93.8±41.8	35.4±8.2	0.0039	0.0053	0.0036	0.0032	0.0126	0.0036	-0.059	-0.057	-0.059	-0.059	-0.046	-0.060	7.628	7.630	7.628	7.629	7.641	7.627
Ag1 Ag7 ot	96*	96*	96*	96*	96*	96*	0.0038	0.0045	0.0036	0.0033	0.0081	0.0035	-0.029	-0.029	-0.029	-0.028	-0.028	-0.028	7.784	7.784	7.784	7.785	7.784	7.784
Ag3 Ag7 ot	96*	96*	96*	96*	96*	96*	0.0053	0.0060	0.0051	0.0048	0.0100	0.0051	-0.035	-0.035	-0.035	-0.034	-0.035	-0.035	7.778	7.777	7.777	7.779	7.778	7.777

	3.0 wt% AgNP	6.0 wt%	8.7 wt%	12.5 wt%	18.6 wt%	24.7 wt%
R-factor	0.009	0.012	0.015	0.018	0.038	0.036
ΔE_0	0.63 ± 0.48	0.59 ± 0.54	0.00± 0.61	0.30 ± 0.65	0.34 ± 0.98	-0.10 ± 0.81

ss: single scattering, at: acute triangle, ot: obtuse triangle, fs: forward scattering, dfs: double forward scattering, fta: forward through absorber. Fixed parameters are reported with a “*”. Amplitude reduction factor S_0^2 was fixed at 0.978 Å. Errors obtained for σ^2 were systematically lower than 0.0035, errors obtained for ΔR and R were systematically lower than 0.0165.

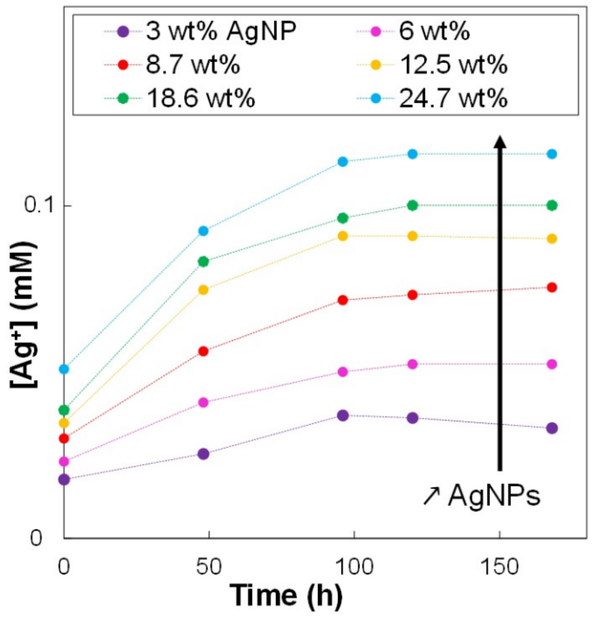

Figure S3. Ag^+ release kinetics over 168h from AgNPs of hybrid NPs in in aqueous medium.

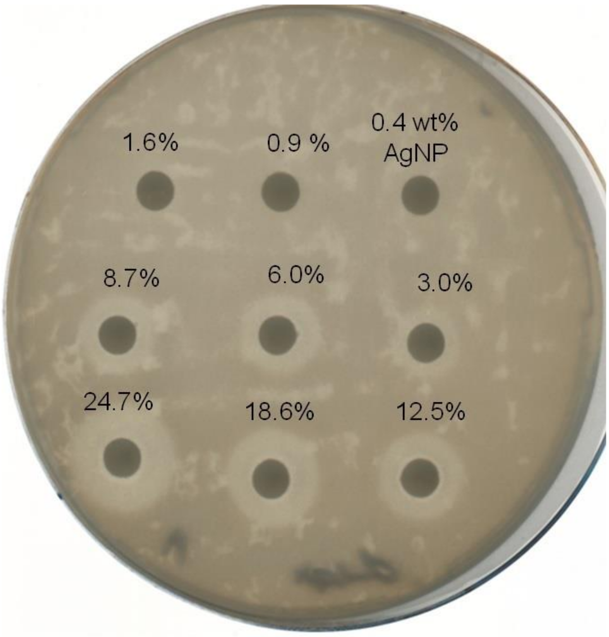

Figure S4. Images of biocide-impregnated paper disks used in diffusion tests for CNC/AgNP hybrids at various AgNP contents.

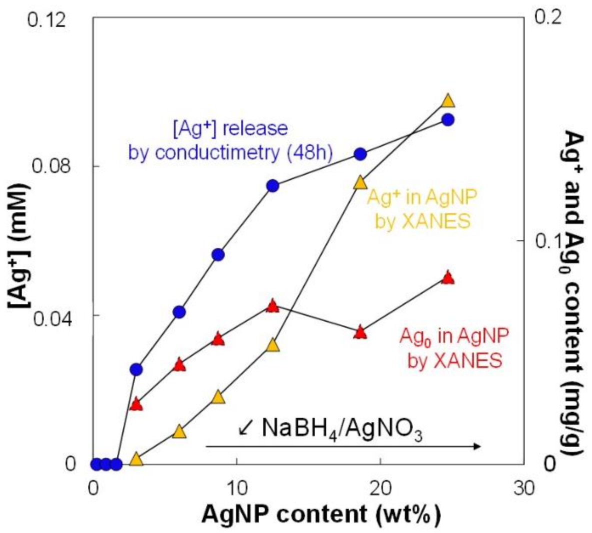

Figure S5. Ag^+ release at 48h in aqueous medium for CNC/AgNP hybrids at various AgNP content estimated by conductimetry.

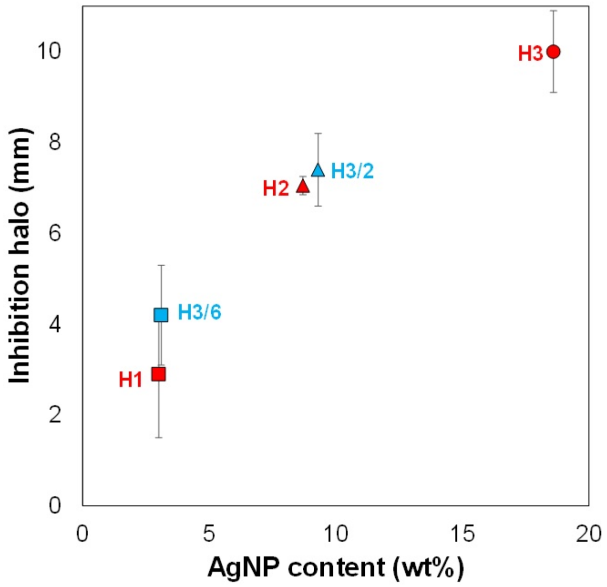

Figure S6. Inhibition halos of hybrid samples used in biocide test to discriminate the antibacterial activity of Ag^+ and Ag^+ fractions in AgNPs.

Table S3. Comparison of MIC in various hybrid systems reported in literature.

Reference	Bacterial strain	MIC (mg AgNP/mL hybrid suspension)	Hybrid suspenson vol. deposited on disk for biocide test (μL)	Ag amount deposed on disk for biocide test (μg AgNP)
**Musino et al.**	*B. subtilis*	0.016	3	0.048
**Shaheen et al.**[1]	*B. subtilis, S. aureus, E. coli, P, aeruginosa*	0.15 – 0.30	100	15 – 30
**Drogat et al.**[2]	*S. aureus, E. coli,*	0.027 – 0.054	100	0.27 – 5.4
**Shi et al.** [3]	*S. aureus, E. coli*	0.04 – 0.08	–	–
**Martinez-Rodriguez et al.**[4]	*E. faecalis*	0.06	50	3
**Caschera et al.**[5]	*E. Coli*	0.11	250	27.5

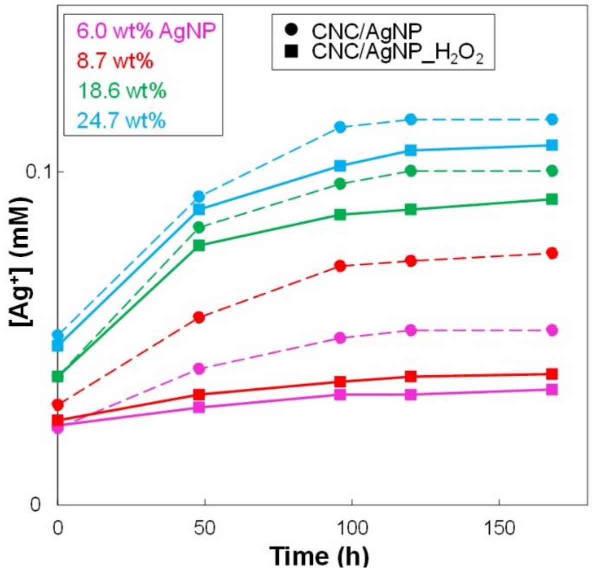

Figure S7. Ag^+ release kinetics monitored by conductimetry in aqueous medium over the time for CNC/AgNP hybrids at various AgNP content with and without H_2O_2 redox post-treatment (i.e., 160 μL H_2O_2).

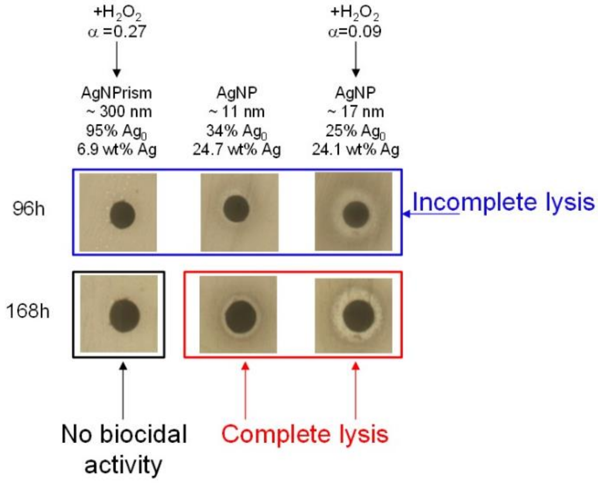

Figure S8. Comparison between long-term diffusion tests for CNC/AgNP hybrids at various AgNP contents with and without H_2O_2 redox post-treatment. Cases of complete and incomplete bacterial inhibition (i.e., lysis) are indicated.

References:

1. Shaheen, T.I.; Fouda, A. International Journal of Biological Macromolecules Green approach for one-pot synthesis of silver nanorod using cellulose nanocrystal and their cytotoxicity and antibacterial assessment. *Int. J. Biol. Macromol.* **2018**, *106*, 784–792, doi:10.1016/j.ijbiomac.2017.08.070.
2. Drogat, N.; Granet, R.; Sol, V.; Memmi, A.; Saad, N.; Klein Koerkamp, C.; Bressollier, P.; Krausz, P. Antimicrobial silver nanoparticles generated on cellulose nanocrystals. *J. Nanoparticle Res.* **2011**, *13*, 1557–1562, doi:10.1007/s11051-010-9995-1.
3. Zengqian Shi, Juntao Tang, a Li Chen, Chuanren Yan, Shazia Tanvir, William A. Anderson, a R.; M. Berry and Kam C Tam Enhanced Colloidal Stability and Antibacterial Performance of Silver Nanoparticles/Cellulose Nanocrystal Hybrids. *J. Mater. Chem. B* **2015**, doi:10.1039/b000000x.
4. Martínez-Rodríguez, M. de los Á.; Madla-Cruz, E.; Urrutia-Baca, V.H.; de la Garza-Ramos, M.A.; González-González, V.A.; Garza-Navarro, M.A. Influence of Polysaccharides' Molecular Structure on the Antibacterial Activity and Cytotoxicity of Green Synthesized Composites Based on Silver Nanoparticles and Carboxymethyl-Cellulose. *Nanomaterials* **2020**, *10*, 1164, doi:10.3390/nano10061164.
5. Caschera, D.; Toro, R.G.; Federici, F.; Montanari, R.; de Caro, T.; Al-Shemy, M.T.; Adel, A.M. Green approach for the fabrication of silver-oxidized cellulose nanocomposite with antibacterial properties. *Cellulose* **2020**, *27*, 8059–8073, doi:10.1007/s10570-020-03364-7.
